# A Novel Mutation in the Adult-Onset Alexander's Disease *GFAP* Gene

**DOI:** 10.1155/2019/2986538

**Published:** 2019-01-10

**Authors:** Dhillon B. Zaver, Nathan T. Douthit

**Affiliations:** Brookwood Baptist Health Medical Education, 817 Princeton Ave SW, POB2, Suite 106, Birmingham, AL 35211, USA

## Abstract

The case describes a 25-year-old Caucasian female diagnosed with Alexander's disease (AxD) as an outpatient after extensive inpatient workup. Her presenting complaints included incontinence, clumsiness, seizures, dysphagia, and dysarthria. She was also found to have pancytopenia and dysautonomia. A full neurologic and hematologic workup yielded very little results, until a thorough literature search of her presenting complaints and radiologic findings pointed to adult-onset Alexander's Disease. Alexander's disease is a rare genetic leukodystrophy with a broad variety of presentations. Despite its infrequency in adults and the difficulty in diagnosis, the prevalence of AxD has been increasing due to ease of genetic analysis and identification of key clinical and radiological findings. This case illustrates the necessity of vigilance and persistence in the face of unusual patient presentations; occasionally, the sound of hoofbeats is zebras.

## 1. Introduction

Alexander's disease (AxD) is a rare genetic leukodystrophy with a broad variety of presentations. Despite its infrequency in adults and the difficulty in diagnosis, the prevalence of AxD has been increasing due to ease of genetic analysis and identification of key clinical and radiological findings. We describe the case of a 25-year-old Caucasian female diagnosed with AxD as an outpatient after extensive inpatient workup.

## 2. Case Presentation

The patient is a 25-year-old Caucasian female who presented to the emergency department after a witnessed event described as a period of rapid eye blinking and unresponsiveness. Her episode ceased after 10 minutes and she complained of numbness and weakness. According to her parents, this was the first event of this description and she was “talking slowly” after the event.

She described incontinence, clumsiness, and difficulty “regulating her body temperature” since middle school. Her past medical history included loss of consciousness after head trauma at age 14 and neuropathic pain in her left arm since a motor vehicle accident at age 20. Later that year, she felt like she “was burning up inside” and collapsed causing a basilar skull fracture. She stated that, prior to losing consciousness, she felt extremely hot but denied diaphoresis. She has had headaches and several similar “burning” episodes without trauma since that time. She then developed dysphagia and dysarthria prompting referrals to ENT, neurology, and psychiatry, leading to diagnoses of anxiety, depression, and sleep apnea. Despite treatment for these diseases, she continued to have episodic hypothermia, weakness, dysphagia, dysarthria, and spasticity. She later went to a traumatic brain injury clinic where she was treated with hyperbaric oxygen and physical therapy. She felt that she had some improvement in that time. At the time of admission, she had worsening symptoms in addition to urinary incontinence, coughing, and spells of unresponsiveness. Social history included avoidance of western medicine and use of several herbal supplements and essential oils daily for health, including colloidal silver. Her family and surgical histories were not significant.

Vital signs on admission included a temperature of 98.7°F, 18 respirations per minute, heart rate of 50 beats per minute, blood pressure of 89/52 mmHg, and oxygen saturation of 96%. Other significant physical exam findings included HEENT exam within normal limits and neurological exam with central lower extremity weakness (3/5) and central upper extremity weakness (4/5). There were bilateral hyperreflexia in the lower extremities and some rigidity with passive movement in the RLE.

Initial laboratory testing was significant for pancytopenia (Table 1). Due to the presenting complaint of seizure, an EEG and MRI were also performed. Initial and extended electroencephalography (EEG) tests were positive for diffuse slowing, indicative of the moderate to severe encephalopathic state of nonspecific etiology. Brain MRI was notable for the diffuse symmetric high T2/FLAIR signal within and along the ependymal lining, involving the subependymal periventricular tissue, raising the possibility of ventriculitis. Other abnormalities included marked wasting and narrowing of upper cervical spinal cord and medullary atrophy as well as increased signal in the pons and cerebellar peduncles. A lumbar puncture was then performed to rule out infection and toxins, but cerebral spinal fluid showed normal cell counts and was negative for herpes simplex virus (HSV), enterovirus, VDRL, cryptococcal antigen, and West Nile virus. Hematologic abnormalities were assessed and ruled out by bone marrow biopsy and flow cytometry.

Lab values, including vitamin levels, serologic studies, thyroid studies, adrenal function, general toxicology screens, and most heavy metal concentrations, were normal. Her blood silver level was elevated to 9.6 mcg/dL (normal is < 5 mcg/dL). This was initially thought to be the cause of the patient's pancytopenia and abnormal MRI findings. Treatment involved maintaining appropriate blood glucose and temperature, initiating a low residue diet and antibiotics, and requesting the family to stop colloidal silver treatment. Her blood counts reached a nadir and recovered during her admission.

Though the pancytopenia resolved with discontinuation of oral silver supplementation, the patient continued to have dysautonomia, dysarthria, and ataxia after discharge. This prompted an investigation into the alternative etiologies that matched her symptoms—specifically the dysautonomia, dysarthria, and ataxia—and unique MRI findings. AxD has been known to cause these symptoms and was consistent with her imaging findings. She was followed up in our resident continuity clinic as an outpatient, where a genetic test for AxD was ordered revealing a missense variation in the glial fibrillation acidic protein (*GFAP*) gene of p.Lys260Gln (c.778 A > C in exon 4 of the *GFAP* gene; K260Q). This was classified as a “likely pathologic variant”; in silico analyses including evolutionary conservation and protein predictors supported a deleterious effect. Her parents were also tested revealing the same mutation in her mother. On further discussion with the mother, she reported decreased balance and coordination that were out of proportion to her age and “difficulty regulating her temperature.” No objective data exist for these complaints, and she has not yet had an MRI performed. There are no other family members with these complaints, although the parents say the patient's younger sister is beginning to show symptoms of dysautonomia. No one else in the family has been officially diagnosed with this disease. She and her family were referred to a local university-affiliated genetic disease clinic for assistance with management.

## 3. Discussion

An extensive workup for the patient's pancytopenia revealed only an elevated silver level as a possible cause. Surprisingly, she had no other known symptoms of silver toxicity, most notably a lack of argyria, a blue-grey color change of mucosal, cutaneous, and conjunctival areas. The potential for absorption and broad deposition in the skin, liver, kidney, and bone is well documented, but the finding of pancytopenia in particular is rare [1]. One case describes that an infant treated with topical silver sulfadiazine for burns over 30% of the body was found to have pancytopenia that eventually reached a nadir and recovered [2]. Leukopenia and bone marrow suppression with silver sulfadiazine in both in vitro and in vivo studies has been shown [3, 4]. Additionally, there have been cases of neurologic symptoms associated with silver toxicity [5, 6]. While some abnormalities in her MRI could be attributable to heavy metal deposition, silver toxicity would not explain her ongoing symptoms; other than pancytopenia, her clinical picture as well as the medullary and cervical spinal atrophy on the MRI were more consistent with AxD.

This diagnosis is generally based on a combination of MRI, clinical, and genomic findings. Van der Knaap et al. have published several studies indicating that the unique MRI findings of AxD, including ventricular and periventricular signal changes or contrast enhancement, brain stem abnormalities including medullary atrophy, and brainstem atrophy, should be used to diagnose or test for AxD. Graff-Radford et al. described many clinical signs of AxD and noted the large variability of symptoms in the adult form of the disease including dysphagia, slurred speech, ataxia, nystagmus, incontinence, hypothermia, gait and sleep disturbances, seizures, and a fluctuating course. The patient described meets the diagnostic MRI criteria and fits the majority of the clinical findings presented earlier, and a genetic sequencing test was done to further solidify the diagnosis.

Using genomic DNA, the coding regions and splice junctions of the requested genes were PCR-amplified, and capillary sequencing was performed. A bidirectional sequence was assembled, aligned to reference gene sequences based on human genome build GRCh37/UCSChg19, and analyzed for sequence variants. Capillary sequencing or another appropriate method was used to confirm all potentially pathogenic variants. If present, apparently homozygous variants were confirmed using alternate primer pairs to significantly reduce the possibility of allele dropout. Sequence alterations were reported according to the Human Genome Variation Society nomenclature guidelines. The missense mutation (K260Q) subsequently found in exon 4 was indicated to likely be deleterious. This was determined using the PROVEAN model with a strong supporting score of −3.49. This particular mutation has not been reported in the Genome Aggregation Database (GNOMAD) or in Clinvar, though nearby missense variations (Y257C, R258C/P, and L264P) have been reported to cause AxD [7]. In our patient, the pathognomonic imaging findings, her clinical support, and the presence of a mutation with deleterious effects in simulation strongly indicates that it is a pathogenic variant.

Alexander's Disease was initially described in infants with failure to thrive, microcephaly, seizures, and rapidly progressive neurological impairment. In the ensuing years, it was determined that this disease could present at any age and have been reclassified into infantile (before two years of age), juvenile (into middle teens), and adult varieties [8]. This leukodystrophy is caused by dominant mutations in the *GFAP* gene and is thought to confer cytotoxicity through gain of function mechanisms [9]. Variable expressivity is commonly mistaken for incomplete penetrance, especially among adult populations [10]. Although the increased availability of *GFAP* testing has made this diagnosis more common, the incidence is still thought to be approximately 1 in 1,000,000 live births [11, 12].

There is a wide range of clinical presentations, but the majority of adult-onset (Type II) AxD patients complain of bulbar signs such as dysphagia and dysphonia, and dysarthria pyramidal signs such as hyperreflexia and ataxia are also common [5, 13]. Dysautonomia, ocular abnormalities, and palatal myoclonus are also not uncommon among Type II AxD patients [8, 9, 11]. Less frequently seen but still present are seizures, sleep disturbances (including apnea and snoring), psychiatric symptoms, and paroxysmal deterioration [8, 11, 14]. Cognitive delay or decline is not a diagnostic feature of Type II AxD [8, 9, 11].

Infantile (Type I) AxD is associated with 5 imaging criteria, including frontal white matter changes; a periventricular rim with high T1 and low T2 signal; T2 hyperintensity involving the basal ganglia and thalamus; brainstem signal abnormalities; and contrast enhancement of particular grey and white matter structures including the ventricular lining, white matter of frontal lobes, optic chiasm, fornix, basal ganglia, thalamus, dentate nucleus, and brainstem [9, 14]. Type II AxD imaging findings are not as well described. Frontal white matter involvement is less common, and brainstem, cerebellar, and spinal cord atrophy predominates [9, 15]. Periventricular hyperintensities are also described [14, 15].

Until 2002, definitive diagnosis was made by brain biopsy revealing Rosenthal fibers [11]. The availability of *GFAP* gene sequencing has increased the frequency of the diagnosis of this rare disorder, but while a number of other mutational abnormalities have been described, the one described earlier has not been proven to cause AxD [8]. Once a pathogenic mutation is discovered, it may be found to be present in family members, some of whom are asymptomatic or minimally affected due to the variable expressivity [10].

Unfortunately, there are no curative therapies for AxD [11, 12]. Most patients with adult-onset disease die at a median age of 25 ± 2.1 years after symptom onset [9]. Management currently involves palliation of symptoms and genetic counseling when needed [12].

Despite this, the cost of a missed diagnosis can still be extremely high. This patient saw several subspecialists, including neurologists, hospitalists, psychiatrist, otorhinolaryngologists, and others, all of whom came to different diagnoses and prescribed different treatments. She also underwent a prolonged hospital stay which did not effectively diagnose or treat any of her problems except the pancytopenia. Finally, the working diagnosis that was communicated to the family was that the patient had residual effects from a traumatic brain injury, had a psychiatric disease, or had her illness caused by their pursuit of complementary and alternative medicines. Having the diagnosis of this progressive neurodegenerative disease will hopefully allow comfort and acceptance of her condition while preventing overly aggressive care of the end of life.

## 4. Conclusion

Adult-onset AxD is a rare genetic leukodystrophy which can present at any age with a wide variation in presentation. For this reason, healthcare practitioners should have a high degree of suspicion for AxD when there are unexplained clinical and imaging findings consistent with the diagnosis. This case describes a new mutation in the *GFAP* gene responsible for AxD based on the clinical findings, imaging findings, and mutation status while illustrating the cost of a missed or delayed diagnosis. The patient experienced unnecessary and overly aggressive interventions, psychosocial stressors, greater use of medical resources, and an almost forced necessity to pursue alternative treatments. It is our hope that awareness of this disease will grow as the diagnostic process becomes better understood and the genetic analysis required becomes more available.

## Figures and Tables

**Table 1:** CBC results tracking pancytopenia throughout admission.

	Admission	Nadir	Discharge
Hemoglobin	8.9 g/dL	6.5 g/dL	9.5 g/dL
Hematocrit	26.9%	19.4%	29.4%
White blood cells	2.1 × 10^9^/L	1.7 × 10^9^/L	5.5 × 10^9^/L
Platelets	76 × 10^9^/L	40 × 10^9^/L	231 × 10^9^/L
